# Supplementary figures and images for: A Humanized Yeast Model for Studying TRAPP Complex Mutations; Proof-of-Concept Using Variants from an Individual with a TRAPPC1-Associated Neurodevelopmental Syndrome
Source: Cells. 2024 Aug 30;13(17):1457. doi: 10.3390/cells13171457 (PMC11394476; doi:10.3390/cells13171457)

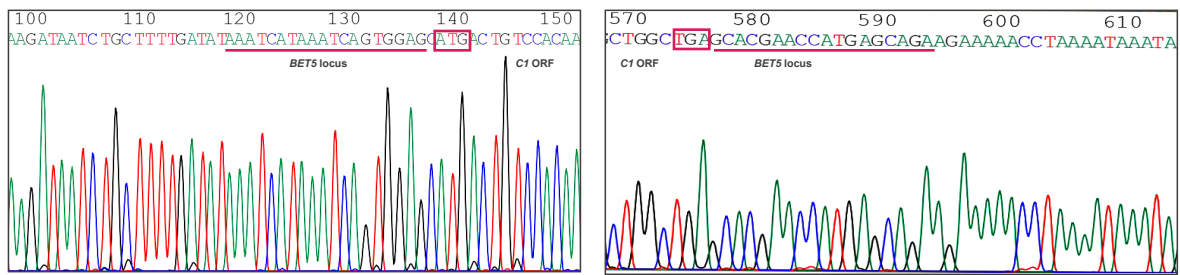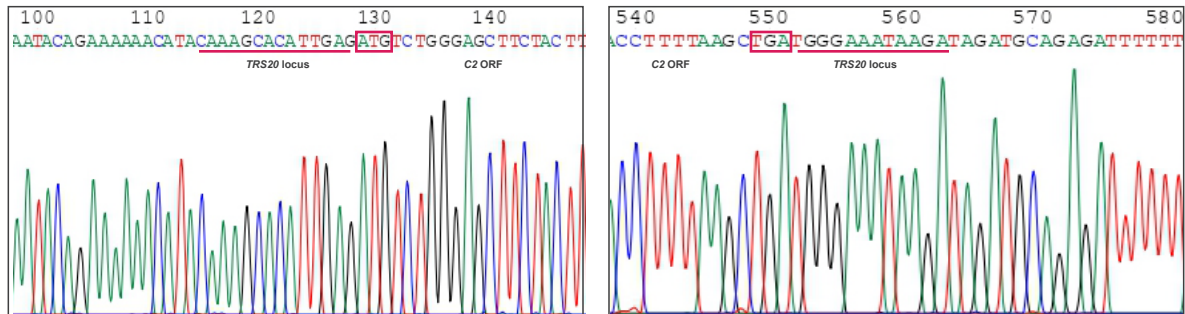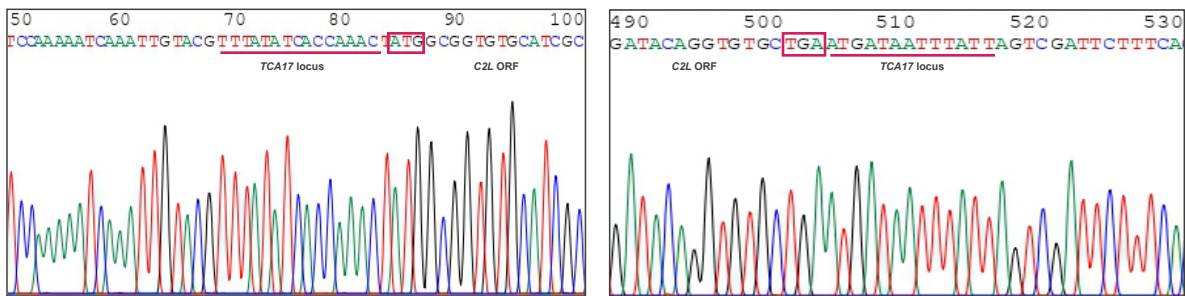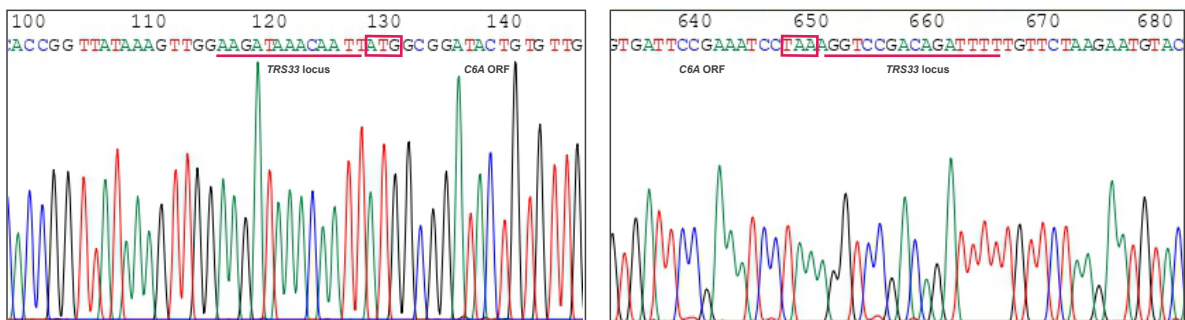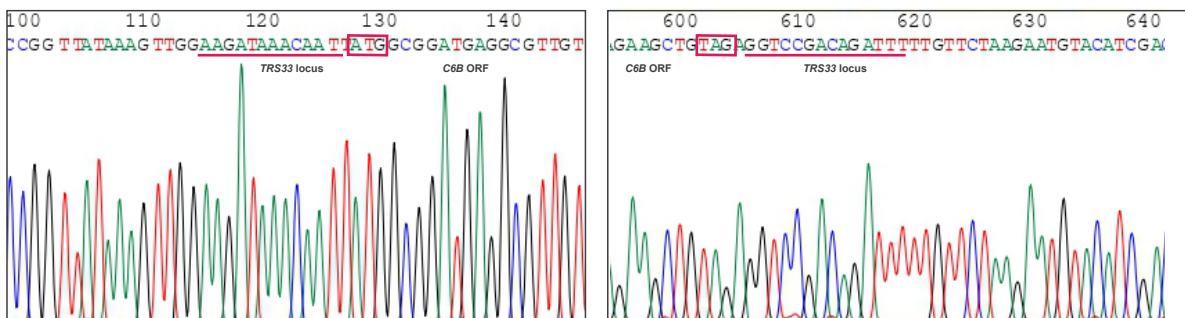

**FIGURE S1**

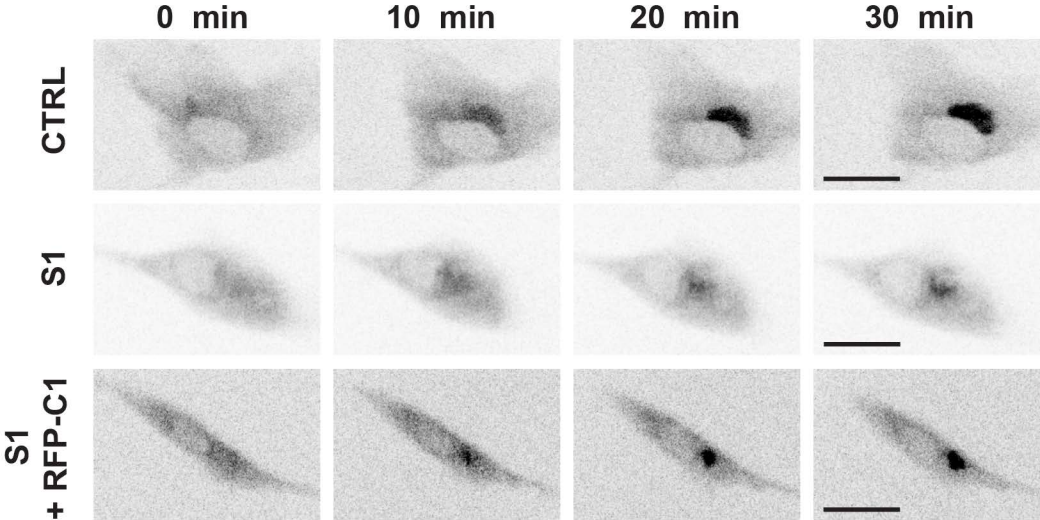

**FIGURE S2**

Supplement: Supplementary file 1 [file cells-13-01457-s001.zip › cells-3167349-supplementary.pdf]
